# Distinctive Characteristics of Rare Sellar Lesions Mimicking Pituitary Adenomas: A Collection of Unusual Neoplasms

**DOI:** 10.3390/cancers17152568

**Published:** 2025-08-04

**Authors:** Andrej Pala, Nadja Grübel, Andreas Knoll, Gregor Durner, Gwendolin Etzrodt-Walter, Johannes Roßkopf, Peter Jankovic, Anja Osterloh, Marc Scheithauer, Christian Rainer Wirtz, Michal Hlaváč

**Affiliations:** 1 Department of Neurosurgery, University of Ulm, Lindenallee 2, 89312 Günzburg, Germany; andreas.knoll@uni-ulm.de (A.K.); rainer.wirtz@bkh-guenzburg.de (C.R.W.); michal.hlavac@uni-ulm.de (M.H.); 2 Endokrinologiezentrum Ulm, Weinbergweg 41, 89075 Ulm, Germany; etzrodtwalter@gmail.com; 3 Department of Neuroradiology, University of Ulm, Lindenallee 2, 89312 Günzburg, Germany; johannes.rosskopf@uni-ulm.de; 4 Department of Neurosurgery, F.D. Roosevelt University Hospital with Policlinic Banska Bystrica, Namestie Ludvika Svobodu 1, 97517 Banska Bystrica, Slovakia; pjankovic@nspbb.sk; 5 Department of Neuropathology, University of Ulm, Lindenallee 2, 89312 Günzburg, Germany; anja.osterloh@bkh-guenzburg.de; 6 Department of Otolaryngology-Head and Neck Surgery, University of Ulm, Frauensteige 12, 89075 Ulm, Germany; marc.scheithauer@uniklinik-ulm.de

**Keywords:** sellar lesions, transsphenoidal pituitary surgery, rare intrasellar neoplasm, intrasellar cyst, endocrine outcome, pituitary gland adenoma

## Abstract

**Background/Objectives**: Pituitary tumors account for over 90% of all sellar region masses. However, a spectrum of rare neoplastic, inflammatory, infectious, and vascular lesions—benign and malignant—can arise in the intra- and parasellar compartments and clinically and radiologically mimic PitNETs. We report a cohort of 47 such rare and cystic midline intracranial lesions, emphasizing their distinctive morphological, clinical, and imaging features and the personalized treatment strategies applied. **Methods**: In this retrospective single-center study, we reviewed all patients treated for suspected PitNETs via transsphenoidal approach between 2015 and 2024. Of 529 surgical cases, we excluded confirmed PitNETs, meningiomas, and classical intradural craniopharyngiomas. Collected data encompassed patient demographics, tumor characteristics, presenting symptoms, extent of resection or medical therapy, endocrine outcomes, and follow-up information. **Results**: Among all 529 patients who underwent surgical treatment for sellar lesions from 2015 to 2024, 47 cases (8.9%) were identified as rare or cystic masses. Forty-six underwent transsphenoidal resection; one patient with hypophysitis received corticosteroid therapy alone. Presenting symptoms included headache (n = 16), dizziness (n = 5), oculomotor disturbances (n = 2), and visual impairment (n = 17). Endocrine dysfunction was found in 30 patients, 27 of whom required hydrocortisone replacement. Histopathological diagnoses were led by colloid cysts (n = 14) and Rathke’s cleft cysts (n = 11). The remaining 22 cases comprised plasmacytoma, germinoma, lymphoma, pituicytoma, inverted papilloma, metastatic carcinoma, chordoma, nasopharyngeal carcinoma, chloroma, and other rare entities. Preoperative imaging diagnosis proved incorrect in 38% (18/47) of cases, with several lesions initially misidentified as PitNETs. **Conclusions:** Nearly 9% of presumed PitNETs were rare, often benign or inflammatory lesions requiring distinct management. Most could be safely resected and demonstrated excellent long-term outcomes. Yet, despite advanced imaging techniques, accurate preoperative differentiation remains challenging, with over one-third misdiagnosed. Clinical red flags—such as early hormone deficits, rapid progression or atypical imaging findings—should prompt early interdisciplinary evaluation and, when indicated, image-guided biopsy to avoid unnecessary surgery and ensure tailored therapy.

## Simple Summary

This study reviews 47 rare and cystic midline lesions that mimicked pituitary adenomas among 529 transsphenoidal surgeries performed from 2015 to 2024. While pituitary adenomas represent over 90% of sellar masses, a spectrum of uncommon neoplastic, inflammatory, infectious, and vascular lesions can present similarly. After excluding classic adenomas, meningiomas, and craniopharyngiomas, we analyzed patient demographics, clinical and imaging features, surgical or medical management, and endocrine outcomes. Forty-six patients underwent surgery; one with hypophysitis received corticosteroids alone. Presenting symptoms included headache, dizziness, oculomotor disturbances, and visual impairment, with 30 patients showing endocrine dysfunction and 27 requiring hydrocortisone. Colloid and Rathke’s cleft cysts were the most frequent diagnoses, alongside 22 other rare entities. Preoperative imaging misdiagnosed 38% of cases. Despite safe resections and favorable outcomes, accurate preoperative differentiation remains challenging; early hormonal deficits, rapid progression, or atypical imaging should trigger interdisciplinary evaluation and potential biopsy.

## 1. Introduction

Pituitary Neuroendocrine Tumors (PitNETs) are the third most common intracranial tumor pathology and a frequent cause of hypopituitarism, visual disturbances, and ophthalmoplegia [1]. Approximately 10% of sellar masses originate from non-pituitary sources [2,3,4,5]. These rare tumors, located in the intra-, supra, and parasellar regions, may mimic PitNETs [6,7] and hinder proper treatment, particularly in the case of rare malignant neoplasms [8,9,10]. We have summarized the essential clinical, radiological, intraoperative, and prognostic characteristics of rare and unusual tumors in the sellar region. We present our findings and experiences with 47 non-adenomatous sellar pathologies identified in a cohort of 529 patients who underwent transsphenoidal surgery (TSS) or conservative treatment. Representative case illustrations and a concise review of each lesion type and its recommended treatment approach are included. Some of these lesions have rarely been reported in the sellar region. This overview aims to assist clinicians and pituitary surgeons in identifying these cases early in the disease process.

## 2. Materials and Methods

### 2.1. Data Collection

This retrospective analysis of all patients (n = 529) was carried out via a transsphenoidal approach at the Department of Neurosurgery at the University of Ulm in Günzburg between 2015 and 2024. All patients with a histopathological diagnosis of PitNET, conventional craniopharyngioma, and meningioma were excluded. Only one special case of extradural craniopharyngioma with a unique osseous feature was included. The remaining 47 patients (8.9%) were included in this analysis. Essential demographic characteristics such as age, gender, initial symptoms, and endocrine function of the pituitary gland were evaluated. Progression-free survival (PFS) was defined as a new tumor-suspected lesion in follow-up Magnetic Resonance Imaging (MRI) or an increase in postoperative tumor remnants in cases of subtotal resection. Endocrinologists evaluated the pituitary function through a multidisciplinary approach. The detailed endocrine evaluation included baseline pituitary hormone levels and hypoglycemic testing to determine cortisol and GH dynamics. Where contraindicated, a CRH test was performed instead. Visual deficits and oculomotor deficits were assessed before and after surgery. The study was conducted according to the International Declaration of Helsinki, and approval from the local ethics committee (Nr. 132/19) was obtained.

### 2.2. Imaging, Histopathological Diagnosis, and Treatment Modalities

All patients’ preoperative MRI scans were evaluated, documenting their characteristics, enhancement patterns, and lesion locations. Imaging included sagittal and coronal T1-weighted sequences, T1-weighted images following gadolinium (Gd) contrast administration, and T2-weighted images. High-resolution computed tomography (CT) scans of the skull base and sellar region were also reviewed in selected cases to enhance the MRI findings. Histopathological analysis was performed at the Department of Neuropathology of Günzburg or the Department for General Pathology at Ulm University. For surgical treatment, two neurosurgeons in our department have performed endoscopic transsphenoidal surgeries using rigid Hopkins (0°, 30°, 45°) endoscopes (Karl Storz, Tuttlingen, Germany) and the four-hand technique. For invasive lesions with skull base infiltration, extended endoscopic approaches were conducted with ENT specialists. A navigation system (Brainlab AG, Munich, Germany) was used in endoscopic and microscopic procedures.

The diagnosis of sellar lesions requires a multidisciplinary approach. Comprehensive evaluations in endocrinology, ophthalmology, and neurology are essential to discuss all treatment options collaboratively. Based on the diagnosis and the presence of residual tumor masses, tailored adjuvant therapy was administered. For suspected pituitary adenoma, the following stains were conducted: reticulin silver staining and immunohistochemistry for synaptophysin, Ki67, and pituitary hormones (ACTH, FSH, LH, hGH, prolactin, TSH).

## 3. Results

**Figure 1:**
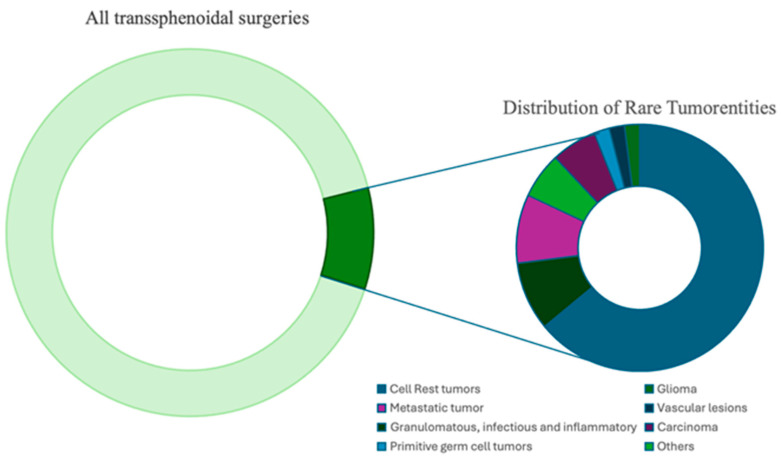
The distribution of rare tumor entities among all transsphenoidal surgeries. The large green donut chart represents the total number of transsphenoidal surgeries (n = 529), with the dark green segment indicating the proportion of rare tumors (8.9%).

In 47 cases, we identified the lesion as a rare or cystic mass. This represents 8.9% of all transsphenoidal procedures (n = 529) performed from 2015 to 2024 (Figure 1). The cohort included 25 women (53%) and 22 men (47%), with a median age of 53 years (range 18–89 years). Most cases were benign (32 out of 47), with a median age of 49 years and a higher prevalence among females (19 out of 32) compared to malignancies (15 out of 47), which had a median age of 61 years and included 9 out of 15 male patients. In this series, six patients (13%) underwent biopsy, three (6%) had partial resection, and radical tumor resection was performed in 37 cases (79%). One patient (2%) with hypophysitis did not have surgery and was managed conservatively.

### 3.1. Histopathological Analysis and Location

The most common diagnosis in this cohort was dysontogenetic tumors, accounting for 30 cases (64%), particularly Rathke’s cleft cysts (23%) and colloid cysts (30%). Most lesions were found in the intrasellar region (42 cases, 89%), followed by the suprasellar region (24 cases, 51%) and the parasellar region (13 cases, 28%). Although combined locations were possible, they were not listed separately. Dysontogenetic tumors were located primarily within the sellar, followed by suprasellar and, rarely, parasellar locations. Pituicytomas, endothelial hemangioendotheliomas, and lymphomas were located at the pituitary stalk, while malignant lesions were often found in the parasellar location. The histopathological results of the entire cohort and anatomical extension in the sellar are summarized in Table 1.

### 3.2. Neurological and Endocrinological Characteristics

Among the rare sellar lesions, dysontogenetic tumors—comprising Rathke’s cleft cysts, colloid cysts, craniopharyngiomas, chordomas, and epidermoid cysts—accounted for the largest group (64%). Despite their shared embryological origin, they presented with distinct clinical profiles. Rathke’s cleft and colloid cysts, which formed the majority, were primarily associated with anterior pituitary insufficiency (64%) and headaches (36%), while visual symptoms were less common. In contrast, craniopharyngiomas and chordomas consistently caused visual impairment (100%) and more frequent cranial nerve involvement. Patients with chordoma, although rare, also showed a high rate of cranial nerve palsy (67%) and visual symptoms. Metastatic lesions (9%) were associated with more severe neurological symptoms, including visual deficits (100%) and cranial nerve palsies (75%), as well as a high rate of endocrine dysfunctions like API (50%) and AVD (25%). Granulomatous and inflammatory lesions (9%) showed a strong association with API (100%) and visual symptoms (75%). Other rare entities, such as carcinomas, germinomas, and lymphomas, frequently cause visual impairment and hormonal deficiencies. Table 2 summarizes all neurological and endocrinological findings of the entire cohort according to their histological diagnosis.

### 3.3. Imaging Characteristics

Most lesions exhibited contrast enhancement, which is often inhomogeneous or marginal, particularly in dysontogenetic and metastatic tumors. Infundibulum involvement was observed in 15 cases, predominantly in dysontogenetic tumors (6 cases), metastatic tumors, pituicytoma, and germinoma. Metastatic tumors were frequently associated with bone destruction, whereas granulomatous and inflammatory lesions display variable contrast enhancement and may involve the chiasm. CT imaging commonly reveals bone destruction and sphenoid sinus infiltration, particularly in aggressive tumors. Of the 47 preoperative radiological diagnoses, 26 (55%) were accurate, 18 (38%) were inaccurate, and three remained unclear. Of the inaccurate preoperative radiological diagnoses, 13 lesions were described as PitNETs or hemorrhagic PitNETs. The lesions most frequently misdiagnosed were colloid cysts (n = 5) and Rathke’s cleft cysts (n = 4), followed by germinoma (n = 1), nasopharyngeal carcinoma (n = 1), necrotic cholesterol granuloma (n = 1), plasmacytoma (n = 1), and extradural craniopharyngioma (n = 1). Notably, cystic lesions overall had the highest diagnostic error rates, particularly when associated with wall thickening or hemorrhagic content, mimicking adenomas on MRI. The AML/chloroma case could not be further specified preoperatively and was radiologically described as a contrast-enhancing mass in the sellar region without a more precise diagnosis. The plasmacytoma, for instance, was radiologically interpreted as a clival chordoma. The craniopharyngioma was misdiagnosed as a cystic fibrous dysplasia. Notably, cystic lesions were the most reliably evaluated across all entities, with 16 out of 25 (64%) correctly assessed preoperatively. Table 3 summarizes all imaging characteristics (MRI and CT).

In 13 cases across the entire cohort, a staging CT of the thorax and abdomen was performed for further evaluation; in 9 cases, the findings were unremarkable. In one instance (an olfactory neuroblastoma), lymph node metastases were detected upon recurrence. In the patient with nasopharyngeal carcinoma, staging revealed lung, liver, and bone metastases (the latter in the context of a second primary prostate cancer), which were already known. Only in the two patients with metastatic tumors did staging detect liver and lung lesions associated with the intrasellar mass.

### 3.4. Treatment and Outcome

The follow-up time ranged from 1 to 144 months, with a mean duration of 25 months. In seven instances, follow-up could not be conducted due to patient decisions. One patient died due to severe complications. Among the patients seen in follow-up, preoperative symptoms improved in 24 patients (51%), remained unchanged in 8 (17%), and were absent in 4 (9%). During the follow-up, we observed a recurrence in four cases (9%), including one colloid cyst, one pituitary abscess, one nasopharyngeal carcinoma, and one olfactory neuroblastoma. Complete resection of colloid cyst as well as complete resection of olfactory neuroblastoma were achieved after primary transsphenoidal surgery. Pituitary abscess was fenestrated and drained completely during the first approach. The patient with nasopharyngeal carcinoma initially underwent biopsy and was treated with pembrolizumab upon local recurrence. The initial presentation involved an extensive lesion with infiltration of the entire skull base.

In comparison, three cases (6%) had a small residual tumor that required no further treatment. One case (2%) remained stable, and three cases (6.4%) achieved remission. The patients with recurrence were treated with repeat surgery (two cases) or managed according to their underlying disease (two cases). Rathke’s cleft cysts (n = 11, 24%) and colloid cysts (n = 14, 30%) were surgically removed using a transsphenoidal approach without radical excision of the cyst wall. The cystic lesions were identifiable intraoperatively. All patients in this cohort with confirmed metastases received postoperative radiation and continued treatment for their primary disease. In the case of the melanoma patient, the dermatologist did not identify a primary lesion, although the staging CT suggested potential liver metastases.

### 3.5. Complications

In this cohort, complications occurred in 11 out of 47 cases (23%). Four instances of rebleeding were recorded—three required coagulations of the sphenopalatine artery, and one necessitated the placement of two external ventricular drains due to intraventricular bleeding, with that patient ultimately succumbing to severe postoperative hemorrhage. Arginine-vasopressin deficiency (AVD) was noted in six patients (13%), all of whom were receiving desmopressin therapy, with two having received it only once. Additionally, cerebrospinal fluid leakage was found in two cases (4%); one was successfully treated with lumbar drainage, and the other had revision surgery. Further analysis showed that complications occurred more frequently in cases with aggressive, infiltrative, or malignant lesions. Specifically, rebleeding and CSF leakage were predominantly associated with lesions showing skull base invasion or firm adherence to surrounding structures, such as metastatic tumors, chordomas, and plasmacytomas. For example, the case with fatal postoperative hemorrhage involved a highly vascular tumor. AVD was more common in patients with inflammatory or infiltrative lesions, such as hypophysitis and lymphoma. In contrast, complications were rare in benign cystic lesions like Rathke’s cleft or colloid cysts, which could be removed more safely via standard transsphenoidal approaches.

## 4. Discussion

In our series, rare non-adenomatous lesions accounted for 8.9% of all intrasellar pathologies—a clinically relevant proportion that warrants heightened diagnostic awareness. In the literature, a prevalence of approximately 10% is reported for rare sellar lesions, which is consistent with our findings [3,8,9]. This incidence might be dependent on the hospital setting and might be lower in primary neurosurgical care. In our high-volume center, some pathologies appeared only once during the study period. Our sample likely does not reflect the true incidence of these entities. The differential diagnosis of rare sellar lesions is extensive, and distinguishing them from PitNETs remains challenging, often requiring histological confirmation [5,9]. Despite advanced imaging techniques, our cohort’s preoperative radiological diagnosis was incorrect in 38% of cases. Treatment of non-adenomatous sellar lesions depends on the underlying pathology, with transsphenoidal surgery (TSS) being the standard approach in most cases. However, rare tumors that mimic PitNETs may infiltrate surrounding structures, posing increased surgical risks. Depending on the pathological findings, additional radiosurgery or specific chemotherapeutic treatment is necessary [8,9,10].

### 4.1. Diagnostic and Therapeutic Considerations

**Figure 2:**
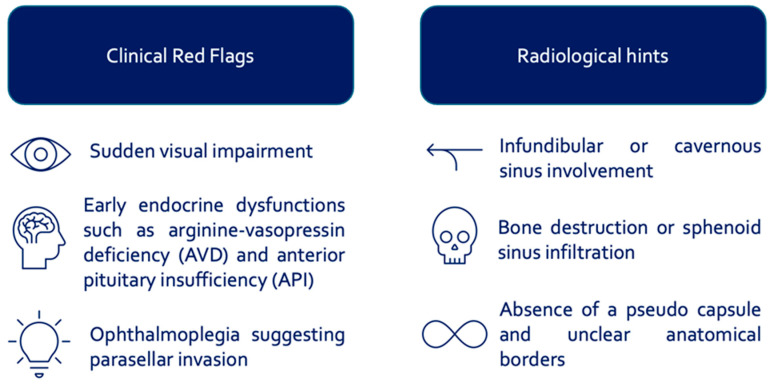
Summary of clinical and radiological differentiation, indicating a rare or malignant lesion.

Differentiating benign from malignant sellar lesions is often difficult, as malignant tumors typically show rapid progression, ophthalmoplegia, and skull base infiltration. The invasive nature of these pathologies does not respect the natural anatomical borders; instead of causing deviation of neurovascular structures, they lead to direct infiltration and invasion. Based on this fact, these tumors are frequently firm and adhesive and pose high surgical risks. Clinically, sudden visual impairment, early endocrine dysfunctions (AVD, API), and ophthalmoplegia indicative of parasellar invasion are key warning signs. Radiologically, infundibular or cavernous sinus involvement, bone destruction or sphenoid sinus infiltration, and the absence of a pseudo-capsule with indistinct anatomical borders should raise suspicion (Figure 2).

Visual impairment was frequently observed in malignant or infiltrative tumors such as chordomas, metastases, and granulomatous lesions. These lesions tend to grow rapidly or invade the optic chiasm directly, as opposed to the compressive mass effect seen in benign PitNETs. Early hormonal dysfunction, particularly API and arginine-vasopressin deficiency, often occurred in patients with hypophysitis, lymphoma, and germinomas. These conditions are characterized by stalk infiltration or autoimmune destruction, resulting in functional loss even in the absence of significant mass effect. Cranial nerve palsies and ophthalmoplegia were strongly associated with lesions invading the parasellar region, especially the cavernous sinus, such as metastatic tumors, chloromas, or osteosarcomas. These tumors often lack a clear surgical plane and show rapid infiltration of surrounding neurovascular structures.

Simple decompression typically does not result in clinical or oncological benefit. The leading differential diagnosis for the acute onset of diplopia is PitNET apoplexy [11]. While benign lesions can usually be safely resected, malignant cases require an individualized surgical strategy. In most instances, a transsphenoidal biopsy is a safe and effective diagnostic tool, enabling targeted therapy with minimal risk. Extensive resection may be considered if the pathology is confirmed, but it must be balanced against potential complications and quality of life. Particularly in older patients or with invasive tumors, limited surgery combined with adjuvant treatment may offer the best outcome.

The primary objectives of transsphenoidal surgery are to achieve secure and efficient diagnostic confirmation, maximize the preservation of pituitary function, and improve visual outcomes. Transsphenoidal optic nerve decompression is a safe procedure associated with excellent clinical outcomes in PitNETs [5,12,13]. This is not always feasible, particularly in malignant parasellar tumors. However, in our series, germinoma resulted in acute visual disturbances, and the resection led to visual improvement. This may be related to the soft consistency of this tumor type, which allowed for indirect decompression. Conversely, the resection of invasive malignant tumors that cause ophthalmoplegia, such as Acute Myeloid Leukemia (AML) or osteosarcoma infiltrating the cavernous sinus, did not result in clinical improvement.

In our cohort, perioperative complications occurred in 23% of cases—a rate significantly higher than that reported in patients undergoing typical transsphenoidal resection of PitNETs. Recent studies show that transsphenoidal surgery for PitNETs has an overall complication rate of around 9–10%, with CSF leaks and meningitis being the most frequent issues. No significant difference was found between microscopic and endoscopic techniques, though endoscopy-specific risks like CSF leaks are influenced by factors such as high BMI, tumor location, and reconstruction technique. Careful surgical planning and modern reconstruction methods significantly reduce complication rates [13,14,15]. Solari et al. reported an overall complication rate of 19% in their analysis of “unconventional” PitNETs, and Somma et al. a complication rate of 28%, consistent with our cohort’s findings [5,11]. These results suggest that atypical sellar lesions are generally associated with a higher risk of complications compared to conventional PitNETs [11].

### 4.2. Differential Diagnoses and Case Presentations

**Figure 3:**
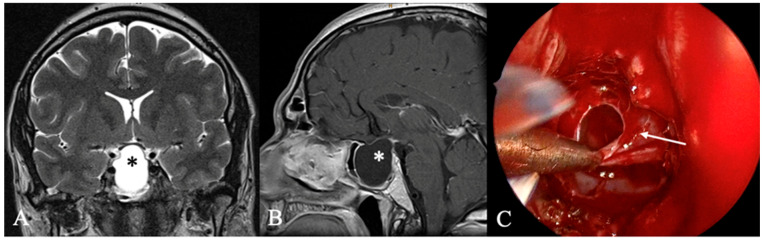
A 67-year-old male patient with a diagnosis of Rathkes cleft cyst. (**A**) T2-weighted and (**B**) contrast-enhanced MRI of a typical Rathke cleft cyst (*). Typical appearance with high T2 and low T1 signal and thin contrast-enhancing capsule. (**C**) Intraoperatively, these cysts appear benign, fluid-filled lesions with a favorable prognosis and a low recurrence rate. The arrow indicates the cyst wall.

Cystic sellar lesions are a frequent differential diagnosis of lesions in the sellar region. They are fluid-filled masses with varying contents, including Cerebrospinal Fluid (CSF), blood, necrosis, or protein-rich fluid, appearing as non-enhancing areas on post-contrast MRI. Enhancement may occur around the cyst wall or residual pituitary tissue. The differential diagnosis of these lesions includes cystic PitNETs, Rathke cleft cysts (Figure 3), craniopharyngiomas, and arachnoid cysts [16,17]. Rathke’s cleft cyst is a benign cystic lesion in the sellar that originates from embryonic remnants of Rathke’s pouch [18]. Symptomatic cases typically appear between 40 and 60 years of age, often when the cysts grow large enough to exert a mass effect on surrounding structures [9,18]. These findings are consistent with our observations. In rare cases, cystic lesions can mimic PitNETs due to wall thickening and chronic inflammation [19].

**Figure 4:**
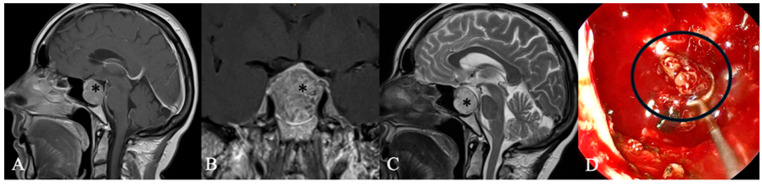
A 59-year-old female patient with the diagnosis of an extradural chordoma. (**A**) Sagittal and (**B**) coronal contrast-enhanced MRI showing a heterogenous contrast-enhancing mass (*) intrasellar. (**C**) T2 Imaging with heterogenous high signal. Bony destruction of the clivus is common. (**D**) Intraoperative findings revealed fibrous intrasellar tissue, more adherent to the cavernous sinus wall than PitNETs, with no pseudocapsule. While tumor resection was feasible, en bloc removal was not possible. The patient returned to work six months after diagnosis, with no recurrence observed 15 months post-surgery.

Chordomas (Figure 4) are rare malignant bone tumors that arise from the embryonic notochord. They grow slowly and display locally aggressive characteristics. Clival chordomas are extradural, exophytic, lytic lesions, and treatment focuses on preserving neurological function. The primary approach involves complete surgical resection, followed by radiation therapy to improve local tumor control. New therapeutic approaches, including targeted therapy and immunotherapy, offer promising potential for improving prognosis [20].

**Figure 5:**
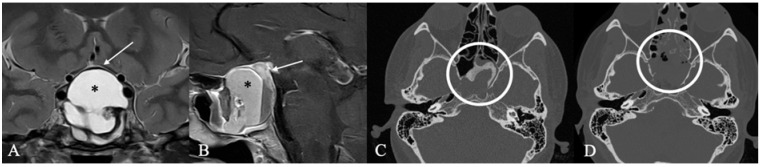
Extradural craniopharyngioma. A 34-year-old female underwent imaging due to persistent headaches. T2 (**A**) and contrast-enhanced T1 (**B**) MRI and CT (**C**) revealed a calcified mass in the sphenoid sinus with cystic extension (*) into the sellar, compressing the optic chiasm (arrow) and pituitary gland (arrow). Histopathology confirmed an unusual case of extradural craniopharyngioma. The tumor, primarily located in the sphenoid sinus, formed an osseous shell that compressed intrasellar structures up to the chiasm. Surgical removal required a high-speed drill while the dura remained intact. (**D**) Postoperative CT scan showed no tumor remnants. The patient recovered fully, and no recurrence was observed 15 months post-surgery.

Craniopharyngiomas (CPs) are rare tumors, accounting for about 1% of central nervous system tumors. Typically, suprasellar, they can extend into the sellar region. While solid-cystic CPs are easily recognized, purely cystic forms pose a diagnostic challenge [16]. In contrast, in our case (Figure 5), the tumor is not only part solid but also part ossified and located in the sphenoid sinus.

**Figure 6:**
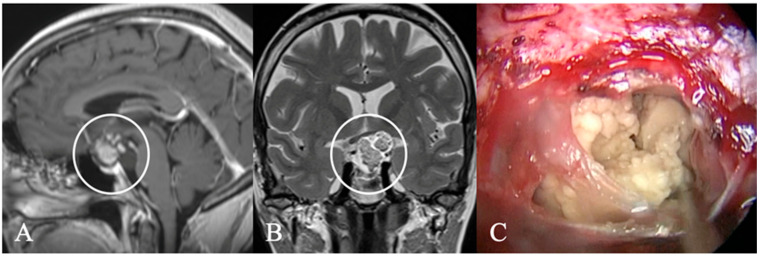
Epidermoid of a 72-year-old female patient. Initially, a visual impairment led to the diagnosis, which regressed immediately after surgery. (**A**) Sagittal contrast-enhanced and (**B**) T2 MRI shows an unusual heterogenous contrast-enhancing intra- and suprasellar mass. Intraoperative imaging (**C**) shows the typical white pearl-like tumor. In our case, complete resection via endoscopic endonasal approach was possible with straightforward reconstruction of the sellar using a fat pad, and no recurrence has been observed five years after the surgery.

Epidermoid cysts (Figure 6) are congenital lesions that arise from neuroectodermal epithelial cells and are usually located in the cerebellopontine angle. Purely sellar tumors are uncommon, with only a handful of reported cases. On MRI, a defining feature of epidermoid cysts is their restricted diffusion, which sets them apart from other cystic tumors [21,22]. Surgical resection is the standard treatment, involving removing the cyst wall to prevent recurrence. The success of the resection primarily relies on the extent of adhesion to parasellar and suprasellar vascular and neural structures. Therefore, the endoscopic endonasal approach seems safe [16,17].

**Figure 7:**
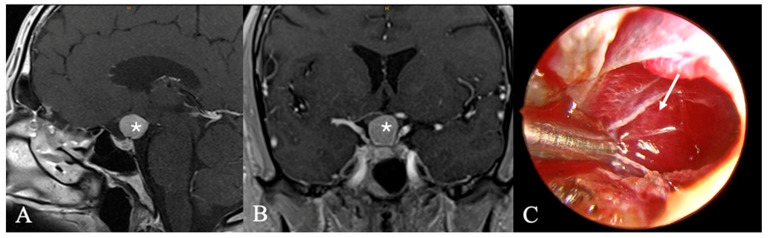
Pituicytoma. In this case of a 57-year-old male patient, secondary hypogonadism prompted imaging studies. Due to the high likelihood of hypopituitarism and arginine-vasopressin deficiency, a biopsy was performed to minimize the risk of complete resection. (**A**,**B**) Sagittal and coronal MRIs reveal a homogeneous contrast-enhanced tumor of the pituitary stalk (*). Intraoperatively (**C**), a firm, compact tumor (arrow) with abundant vasculature infiltrating the pituitary stalk was noted. Thus far, 18 months post-surgery, no new symptoms or tumor progression have been observed.

Pituicytomas (Figure 7) are rare tumors from glial cells called pituicytes, localized in the infundibulum or the neurohypophysis and seen with a mean age of 40 years with a male dominance [23,24,25], in accordance with our case. This highly vascular tumor may present with spontaneous hemorrhage, and its dense capillary network can lead to significant intraoperative bleeding, often restricting complete surgical removal [26]. MRI features of pituicytoma are nonspecific. They show a solid, well-defined sellar or suprasellar mass, typically isointense on T1 and hyperintense on T2-weighted sequences, with usually no cystic components [24,27,28].

**Figure 8:**
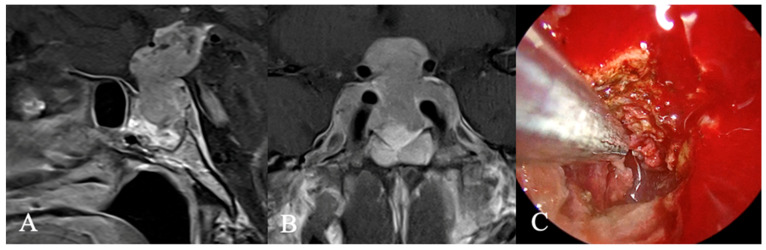
Metastatic tumor lung cancer of a 74-year-old male patient. (**A**,**B**) Sagittal and coronal contrast-enhanced MRI reveal an intrasellar and suprasellar tumor infiltrating the surrounding tissue. (**C**) Intraoperative findings confirmed the presence of firm, tightly adherent tissue with a high risk of collateral injury. The well-vascularized tumor invaded both parasellar and suprasellar structures, making gross total resection rarely feasible due to the risk of vascular damage.

Pituitary and sellar metastases are rare, occurring in fewer than 1% of transsphenoidal surgeries for sellar or parasellar tumors, with breast, prostate, and lung cancer being the most common causes [9,22,29,30]. The AVD is discussed to be a leading symptom in metastatic tumors [29,30]. In our cohort, the patients with metastatic tumors (Figure 8) underwent a transsphenoidal biopsy and partial resection due to firm consistency and adherence to vascular structures. During staging examinations, both patients had more than one suspect finding, which indicates an advanced stage of the disease.

**Figure 9:**
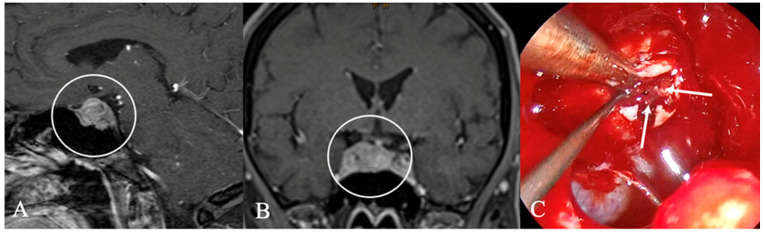
Acute Myeloid Leukemia (AML) chloroma. A 77-year-old male underwent imaging due to oculomotor palsy. (**A**,**B**) Sagittal and coronal contrast-enhanced MRI showed a heterogenous enhancing mass, misinterpreted as PitNET. Endocrinological examination revealed additional anterior pituitary insufficiency requiring hydrocortisone therapy. Intraoperatively, the tumor appeared atypical, firm, and difficult to separate from intrasellar structures, lacking clear dissection planes (**C**). Gross total resection was high risk due to the potential vascular injury, which made it impossible. Postoperatively, the patient received adjuvant radiation and continued treatment under internal medicine. Clinically, his condition remained unchanged after surgery.

Chloroma (Figure 9) signifies the extramedullary proliferation of immature myeloid precursors observed in various myeloproliferative and myelodysplastic disorders, most commonly in acute myeloid leukemia [31].

**Figure 10:**
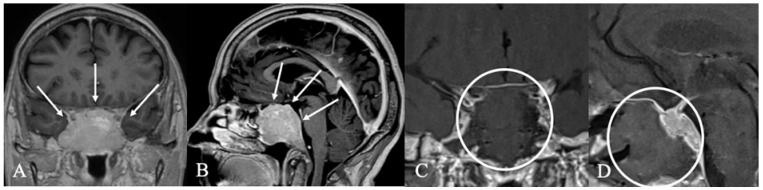
Plasmocytoma. A 78-year-old female patient presented with facial nerve palsy, trigeminal nerve involvement, decreased visual acuity, and anterior pituitary insufficiency, which was treated with hydrocortisone. Initial contrast-enhanced MRI (**A**,**B**) raised suspicion of a clival chordoma; however, histological analysis confirmed plasmacytoma. Intraoperatively, significant bony and mucosal infiltration was noted. Postoperatively, she received skull base radiotherapy with a total dosage of 36 Gy. No further therapy was necessary since the SLiM CRAB criteria [36] were unmet. Her symptoms improved after surgery, and her functional status recovered. Images (**C**,**D**) illustrate the extent of resection. Thirteen months post-surgery, the disease remains stable.

Plasmacytoma (Figure 10) of the skull base is an exceedingly rare tumor, with only a handful of cases reported in the literature. The most frequently affected site is the nasopharynx, accounting for 18% of all head and neck cases, while less common presentations involve the sphenoid, clivus, and petrous apex [32,33,34]. Known independent factors predicting the outcome are younger age and tumors smaller than 5 cm [35].

**Figure 11:**
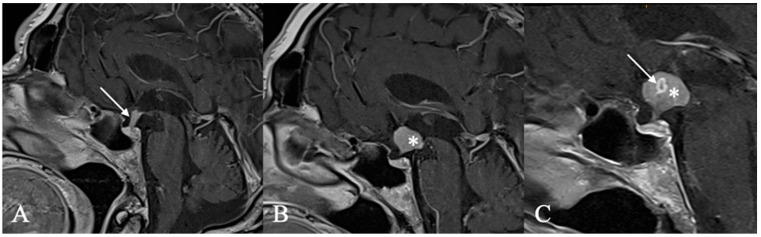
B-cell-lymphoma. A 64-year-old male presented with acute arginine-vasopressin deficiency and hypopituitarism. (**A**) Sagittal contrast-enhanced MRI showed a thickened pituitary stalk (arrow). Hormonal replacement therapy initially led to clinical improvement, but within three months (**B**), rapid tumor progression (*) was observed, followed by central necrosis (arrow, **C**) one month later. A transventricular endoscopic biopsy revealed soft, vascularized tissue, and histopathology confirmed a diagnosis of diffuse large B-cell lymphoma. Staging CT and spinal MRI showed no signs of metastases. Four months after surgery and adjuvant therapy, the patient remains in remission.

The sellar region’s primary lymphoma (Figure 11) is extremely rare, with only a few reported cases in patients aged 44 to 86. Symptoms are nonspecific and may include headaches, hypopituitarism, visual field deficits, and oculomotor palsies. MRI features are largely indistinct, typically exhibiting homogeneously or heterogeneously enhanced sellar masses. T2-weighted imaging often shows iso- to hypointense signals compared to gray matter, which may assist in differentiating it from PitNETs [9,37,38].

**Figure 12:**
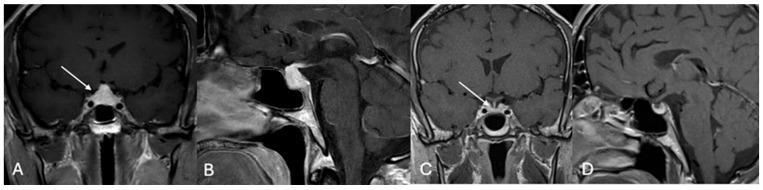
Hypophysitis. In this case of hypophysitis of a 40-year-old male patient, surgery was avoided due to an accurate endocrinological diagnosis. The patient received high-dose corticosteroid therapy, leading to complete disease resolution. (**A**,**B**) Coronal and sagittal MRIs show an enlarged contrast-enhancing pituitary gland and prominent enhancing pituitary stalk; these findings initially made it suspicious to diagnose a PitNET. (**C**,**D**) MRI follow-up after 15 months showed regression, and hormone replacement therapy was not required. This highlights the importance of precise diagnosis and close collaboration between endocrinologists, radiologists, and neurosurgeons in managing such cases without surgical intervention.

**Figure 13:**
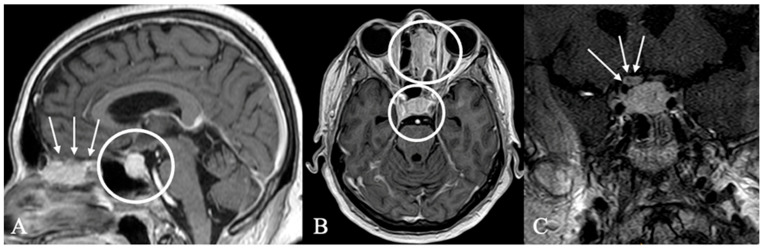
A 60-year-old female patient with a diagnosis of granulomatous inflammation. (**A**,**B**) Sagittal and axial contrast-enhanced MRIs show a contrast-enhancing mass in the sellar and paranasal sinuses (circle, arrows). (**C**) Coronal FLAIR MRI shows a contrast enhancement of the right optic nerve. Lumbar puncture revealed signs of an inflammatory process. Intraoperatively, the lesion showed yellowish soft granules that were easy to dissect and biopsy. There was no relevant vasculature or bleeding risk.

**Figure 14:**
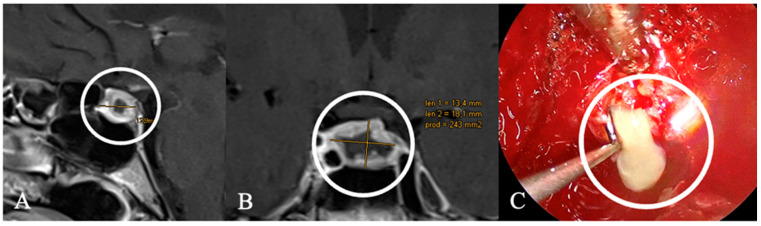
Pituitary abscess of a 61-year-old female patient. (**A**,**B**) Sagittal and coronal contrast-enhanced MRI show a contrast-enhancing intrasellar mass, with the center being necrotic. Three transsphenoidal surgeries (TSSs) for pus drainage ((**C**), intraoperative impression) were required, as antibiotic therapy alone was insufficient. Despite initial conservative management of meningitis, the abscess recurred, necessitating a second surgery. Over a year later, a third procedure, including partial hypophysectomy, was performed due to worsening visual disturbances and progressive hemianopsia. Cultures remained negative, and the patient received long-term meropenem treatment. After surgery and appropriate management of hypopituitarism, symptoms resolved, with significant improvement observed at the 24-month follow-up.

Autoimmune hypophysitis (AH) (Figure 12) and infections (Figure 13 and Figure 14) are rare diseases and are misdiagnosed in 40% [19,39]; however, they usually relate to severe symptoms of hypopituitarism and can become life-threatening due to acute Addison’s crisis or severe meningitis in cases of bacterial infection (Figure 14) [19]. MRI shows that AH may present diverse features, most commonly a thickened, non-deviated stalk. Over 80% also show mild to moderate symmetric gland enlargement, which can lead to misreading the MRI and may lead to the diagnosis of a PitNET [19,39,40].

**Figure 15:**
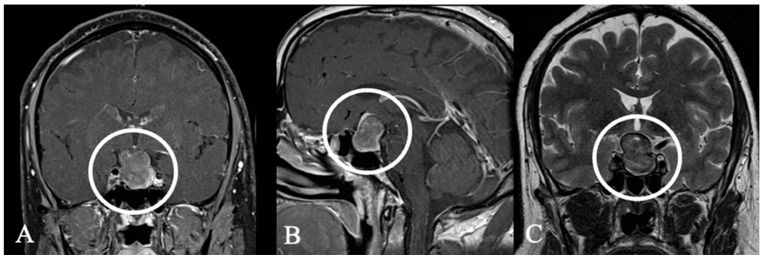
Necrotic cholesterol granuloma. A 51-year-old male patient presented with headaches and adynamia at initial diagnosis, along with reduced vision in the right eye. Endocrinological evaluation confirmed anterior pituitary insufficiency and arginine-vasopressin deficiency (AVD). (**A**,**B**) Coronal and sagittal contrast-enhanced MRI shows an inhomogeneous contrast-enhancing mass in the sella with low inhomogeneous signal in the T2 sequence (**C**). Radiologically, a hemorrhagic PitNET was diagnosed. Intraoperatively, the mass appeared as waxy, xanthochrome tissue. Complete resection was achieved.

Sellar cholesterol granulomas (Figure 15) are rare lesions, more common in females. They typically appear as T1 hyperintense lesions on MRI and can mimic craniopharyngiomas or Rathke’s cleft cysts. They are associated with younger age and more frequent, severe endocrinological deficits. Recurrence is uncommon, even in cases of subtotal resection [41,42].

**Figure 16:**
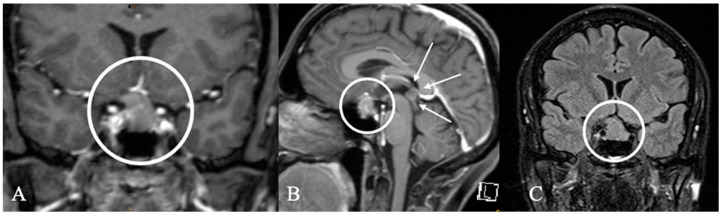
Germinoma. Acute visual disturbances in a 39-year-old male patient led to an MRI (**A**–**C**), which detected a mass suspicious for a PitNET. The findings prompted urgent chiasm decompression via TSS. In retrospect, an enlargement of the glandula pinealis was confirmed ((**B**), arrow). Post-surgery, the patient’s vision improved, and histopathology confirmed germinoma. Notably, common symptoms such as arginine-vasopressin deficiency, hyperprolactinemia, or hypopituitarism were absent. Serum and CSF tumor markers were negative. The craniospinal axis MRI was without metastases.

Intracranial germ cell tumors are midline lesions that typically occur in the pineal or suprasellar regions. Primary intrasellar germinomas are rare [22,43]. Serum and CSF tumor markers, such as AFP or β-HCG, are crucial in diagnosing germinomas. Because germinomas (Figure 16) tend to spread via CSF, MRI of the craniospinal axis and lumbar puncture are essential. Pure germinomas are radiosensitive and have a favorable prognosis [9].

**Figure 17:**
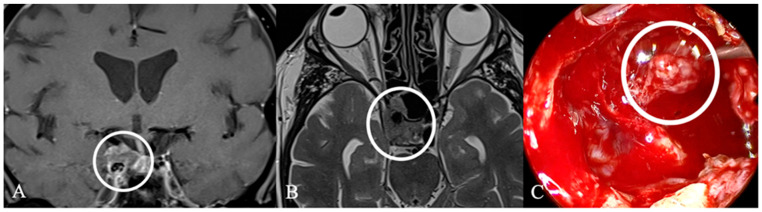
Carcinoma. A 72-year-old male. (**A**) Coronal contrast-enhanced MRI with inhomogeneous enhancement and (**B**) axial T2 images show a parasellar mass with intracavernous tumor growth encasing the internal carotid artery. (**C**) Intraoperative image. Histopathological analysis following biopsy confirmed nasopharyngeal carcinoma. After the confirmed diagnosis, skull base radiation and tailored chemotherapy were performed. At 48 months post-diagnosis, the patient is in remission.

Nasopharyngeal carcinoma (NPC) (Figure 17) is a malignant epithelial tumor strongly associated with Epstein–Barr virus infection, genetic susceptibility, and environmental factors, with a high prevalence in southern China and Southeast Asia. According to Han et al., intrasellar involvement in NPC occurs predominantly through direct invasion (57.2%), whereas true metastasis to the sellar is exceedingly rare, occurring in approximately 1% of cases [44].

**Figure 18:**
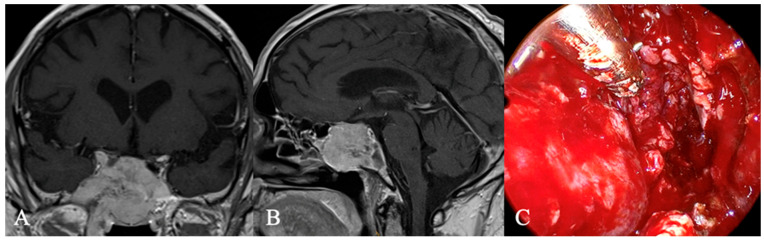
High-grade osteosarcoma. A 75-year-old male patient with high-grade osteosarcoma. (**A**) Coronal and (**B**) sagittal contrast-enhanced MRI demonstrate an extensive contrast-enhancing mass with destructive growth pattern along the skull base with the complete pituitary gland and infundibulum encasement. (**C**) Intraoperative findings revealed firm, well-vascularized tissue infiltrating surrounding structures without clear borders, making gross total resection challenging due to skull base involvement. Postoperatively, he received 50 Gy of radiotherapy and chemotherapy with doxorubicin and gemcitabine.

Osteosarcoma of the skull base (Figure 18) is extremely rare, and the literature is limited to individual case reports. The optimal management approach combines radical surgical resection with comprehensive multimodal therapy [45].

**Figure 19:**
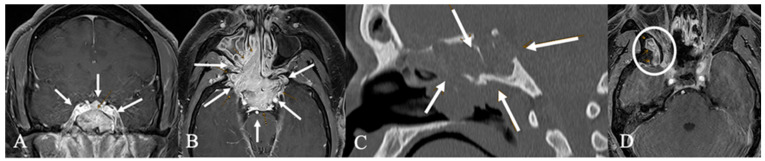
Inverted papilloma. A 27-year-old male presented with nasal obstruction. (**A**,**B**) Contrast-enhanced MRI revealed a significant skull base and intranasal tumorous lesion, suspicious for inverted papilloma, which was histopathologically confirmed later. He exhibited no visual impairments or endocrine deficits either before or after surgery. Intraoperatively, destructive growth with osseous infiltration (**C**) was observed. A bilateral trans-nasal endoscopic pan sinus surgery was performed with complete visualization of the frontal base. The postoperative MRI (**D**) reveals a small residual lesion on the right sphenoid wing.

Inverted papilloma (IP) (Figure 19) is a benign epithelial tumor originating from the sinonasal cavities. It is known for its local aggressiveness, frequent recurrence, and possible association with carcinoma. The incidence is 0.2–1.5 per 100,000 per year, with a male-to-female ratio of 2–5:1 and an average diagnosis age of 55. Surgical treatment relieves symptoms and allows complete pathological evaluation [46,47,48].

**Figure 20:**
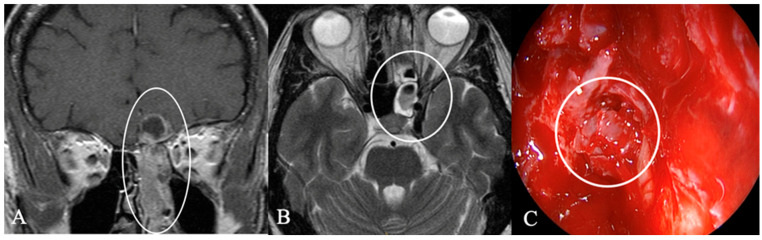
Olfactory neuroblastoma. A 57-year-old female presented with nasal breathing obstruction. (**A**) Coronal contrast-enhanced MRI and (**B**) T2 MRI reveal a heterogeneous, partly cystic tumorous lesion involving the sellar region and paranasal sinuses. (**C**) Intraoperative image demonstrating infiltrative growth. Postoperatively, radiotherapy (up to 54 Gy) was administered. Seventy-two months later, submandibular metastases and a diffuse cerebral, dural spread were observed.

Olfactory neuroblastoma (ONB) (Figure 20) is a rare malignant neoplasm originating from olfactory receptor cells in the superior nasal vault. It accounts for 2–3% of nasal cavity tumors and has an incidence of 0.4 cases per million [49,50]. It affects both sexes across a wide age range (2–90 years) and is best treated with endoscopic surgical resection followed by postoperative radiation therapy [49,50,51,52].

## 5. Limitations

This cohort includes many tumors and lesions, many of which are represented by only a single case. Due to the small sample size, statistical analysis is not feasible, and the findings are purely descriptive. Furthermore, our study’s retrospective design and monocentric nature are potential biases in our evaluation. There is also a potential selection bias, as our neurosurgical department is a referral center. This may have led to a relative overrepresentation of rare, complex, or advanced sellar pathologies, particularly those requiring surgical treatment. In addition, only histologically confirmed or biopsy-proven lesions were included, potentially excluding milder or non-surgical cases. Furthermore, the relatively long study period (2015–2024) may have introduced subtle variability in imaging protocols or surgical strategies.

## 6. Conclusions

Accurate recognition of rare non-adenomatous sellar lesions is essential for guiding optimal treatment, including surgical and adjuvant therapies. Differentiating these lesions from PitNETs remains challenging due to overlapping clinical and radiological features. Subtle imaging clues and clinical red flags—such as rapid growth, early hormonal deficits, or sudden neurological symptoms—may indicate an atypical pathology and encourage clinicians to consider different approach, as in the case of classical PiTNETs, such as transsphenoidal biopsy, which is safe and allows for a rapid histopathological evaluation. Most lesions, such as Rathke cleft cysts, are benign with excellent prognoses and can be safely resected using appropriate surgical techniques. In benign or soft tumors, decompression of the optic chiasm and cavernous sinus is often effective and associated with good outcomes. In contrast, malignant perisellar tumors often lead to persistent ophthalmoplegia, hypopituitarism, or visual deficits due to their invasive nature. Aggressive resection may result in severe complications without providing any benefit in terms of neuro-oncological quality of life balance. Further studies, such as register studies are necessary to improve the knowledge and characteristics of rare perisellar pathologies as well as its therapy in the future.

## Figures and Tables

**Table 1:** The histopathological classification and anatomical extension of rare sellar lesions based on 47 patients. Most lesions showed an intrasellar extension (89%). Certain tumors, such as chordomas, epidermoids, craniopharyngiomas, metastases, and carcinomas, typically extend across all three regions. In contrast, Rathke’s cleft and colloid cysts were mainly limited to the intrasellar and suprasellar areas. Inflammatory and infectious lesions also predominantly showed intrasellar localization. Entities like germinoma, pituicytoma, and pituitary abscesses were each seen in only one patient and were confined to specific regions, such as the intrasellar space or pituitary stalk.

Histopathological Diagnosis	No. of Patients	Extension of Sellar Lesions		
	% (n)	Suprasellar % (n)	Intrasellar % (n)	Parasellar % (n)
Dysontogenetic tumors, total Rathke’s cleft cyst Craniopharyngioma (extradural) Chordoma Epidermoid Colloid cyst	64% (30) 23% (11) 2% (1) 6% (3) 2% (1) 30% (14)	60% (18) 64% (7) 100% (1) 100% (1) 64% (9)	97% (29) 100% (11) 100% (1) 100% (3) 100% (1) 93% (13)	13% (4) 100% (1) 100% (3)
Pituicytoma	2% (1)	100% (1) Pituitary stalk		
Metastatic tumor, total Amelanotic melanoma Chloroma Plasmocytoma Bronchial Carcinoma	9% (4) 2% (1) 2% (1) 2% (1) 2% (1)	50% (2) 100% (1) 100% (1)	100% (4) 100% (1) 100% (1) 100% (1)	75% (3) 100% (1) 100% (1) 100% (1)
Vascular lesions, total Endotheliod hemangioendothelioma	2% (1) 2% (1)	100% (1) Pituitary stalk		
Granulomatous, infectious and inflammatory, total Granulomatous inflammation Pituitary abscess Hypophysitis Necrotic cholesterin granuloma	9% (4) 2% (1) 2% (1) 2% (1) 2% (1)	25% (1) 100% (1)	100% (4) 100% (1) 100% (1) 100% (1) 100% (1)	
Carcinoma, total Nasopharyngeal High-grade osteosarcoma	6% (3) 4% (2) 2% (1)		100% (3) 100% (2) 100% (1)	100% (3) 100% (2) 100% (1)
Germ cell tumors, total Germinoma	2% (1) 2% (1)		100% (1) 100% (1)	
Others Lymphoma Inverted papilloma Olfactory neuroblastoma	6% (3) 2% (1) 2% (1) 2% (1)	100% (1) Pituitary stalk	100% (1) 100% (1)	100% (1) 100% (1)
Total	100% (47)	51% (24)	89% (42)	23% (11)

**Table 2:** Neurological and endocrinological symptoms of patients with rare sellar lesions before treatment. Visual impairment includes both reduced visual acuity and visual field deficits.

		Neurology			Endocrinology	
	No. of Patients % (n)	Headache % (n)	Visual Impairment % (n)	III, IV, V Palsy % (n)	API % (n)	AVD % (n)
Dysontogenetic tumors Rathke’s cleft cyst and colloid cyst Craniopharyngioma Chordoma Epidermoid	64% (30) 53% (25) 2% (1) 6% (3) 2% (1)	37% (11) 36% (9) 100% (1) 100% (1)	20% (6) 12% (3) 100% (1) 33% (1) 100% (1)	7% (2) 67% (2)	57% (17) 64% (16) 33% (1)	10% (3) 12% (3)
Pituicytoma	2% (1)		100% (1)		***	
Metastasis	9% (4)	25% (1)	100% (4)	75% (3)	50% (2)	25% (1)
Vascular lesions	2% (1)		100% (1)		100% (1)	
GII	9% (4)	50% (2)	75% (3)		100% (4)	50% (2)
Carcinoma	6% (3)	33% (1)	33% (1)	67% (2)	33% (1)	
Germinoma	2% (1)		100% (1)			
Others: Lymphoma Inverted papilloma Olfactory neuroblastoma	6% (3) 2% (1) 2% (1) 2% (1)	33% (1) 100% (1)			33% (1) 100% (1)	33% (1) 100% (1)
Total	100% (47)					

GII: Granulomatous, infectious, and inflammatory, Others: Lymphoma, IP, ONB, API: anterior pituitary insufficiency, AVD: Arginine-vasopressin deficiency. IP: Inverted papilloma. ONB: Olfactory neuroblastoma. *** secondary hypogonadism as the single pituitary disfunction.

**Table 3:** Imaging characteristics of patients with rare sellar lesions.

	MRI			CT	
Tumor Entity % (n)	Contrast enhancement % (n)	Infundibulum % (n)	Chiasma % (n)	Bone destruction % (n)	Sphenoid sinus infiltration % (n)
Dysontogenetic tumors 64% (30)	43% (13) inhomogeneous 47% (14) marginal	20% (6) infiltrated	37% (11) contact 13% (4) compression	10% (3) osteolytic 7% (2) infiltrated	17% (5)
Pituicytoma 2% (1)	100% (1) homogeneous	100% (1) infiltrated	100% (1) compression		
Metastasis 9% (4)	75% (3) inhomogeneous 25% (1) homogeneous	75% (3) infiltrated	75% (3) compression	75% (3) osteolytic	75% (3)
Vascular 2% (1)	100% (1) inhomogeneous	100% (1) infiltrated	100% (1) contact		
GII 9% (4)	25% (1) inhomogeneous 50% (2) homogeneous 25% (1) marginal	25% (1) infiltrated	75% (3) contact 25% (1) compression	25% (1) osteolytic	25% (1)
Carcinoma 6% (3)	33% (1) inhomogeneous 67% (2) homogeneous			100% (1) infiltrated	66% (2)
Germinoma 2% (1)	100% (1) inhomogeneous	100% (1) infiltrated	100% (1) compression		
Others: 6% (3)	100% (3) inhomogeneous	33% (1) infiltrated		66% (2) osteolytic	66% (2)
Total: 100% (47)					

## Data Availability

The data presented in this study are available on request from the corresponding author.

## Abbreviations

The following abbreviations are used in this manuscript:

AH	Autoimmune Hypophysitis
API	Anterior Pituitary Insufficiency
AML	Acute Myeloid Leukemia
AVD	Arginine-Vasopressin deficiency
BD	Bone Destruction
CP	Craniopharyngiomas
CO	Chiasma Opticum
CSI	Cavernosus Sinus Infiltration
CT	Computed Tomography
CSF	Cerebrospinal Fluid
D	Dizziness
ENT	Ear Nose Throat
EoR	Extent of Resection
ES	Extrasellar
Gd	Gadolinium
GII	Granulotaous Infectious Inflammatory
HA	Headache
I	Infundibulum
MR	Magnetic Resonance Imaging
NPC	Nasopharyngeal Carcinoma
ONB	Olfactory Neuroblastoma
IS	Intrasellar
IP	Inverted Papilloma
PitNET	Pituitary Neuroendocrine Tumor
PS	Parasellar
PFS	Progression-free survival
SS	Suprasellar
SH	Secondary Hypogonadism
SSI	Sphenoid Sinus Infiltration
VI	Visual Impairment
